# Fruit Leathers: Method of Preparation and Effect of Different Conditions on Qualities

**DOI:** 10.1155/2014/139890

**Published:** 2014-05-04

**Authors:** Lemuel M. Diamante, Xue Bai, Janette Busch

**Affiliations:** Department of Wine, Food and Molecular Biosciences, Lincoln University, Canterbury, Lincoln 7647, New Zealand

## Abstract

Fruit leathers are dehydrated fruit products which are eaten as snacks or desserts. They are flexible sheets that have a concentrated fruit flavor and nutritional aspects. Most fruit leathers are prepared by mixing fruit puree and other additives like sugar, pectin, acid, glucose syrup, color, and potassium metabisulphite and then dehydrating them under specific conditions. Various drying systems including combined convective and far-infrared drying, hot air drying, microwave drying, solar drying, and sun drying have been used to make fruit leathers. Most fruit leathers are dried at 30 to 80°C for up to 24 hours until the target final moisture content (12–20%) has been reached. Research about fruit leathers began in the 1970s. This work has reviewed published papers on fruit leathers in order to summarize useful information about fruit leathers on methods of preparation, effects of drying condition, and effects of packaging and storage, which will be useful to many in the food industry and consumers who are health-conscious.

## 1. Introduction

Fruit leather, also called a fruit bar or a fruit slab, is a dehydrated fruit-based confectionery dietary product which is often eaten as snack or dessert [1]. It is chewy and flavorful, naturally low in fat and high in fiber and carbohydrates; it is also lightweight and easily stored and packed [2]. Consuming fruit leather is an economic and convenient value-added substitute for natural fruits as a source of various nutritional elements. Furthermore, fruit leather has far fewer calories, ≤ less than 100 kcals per serving, than many other snacks [3]. Fruit leathers are restructured fruit made from fresh fruit pulp or a mixture of fruit juice concentrates and other ingredients after a complex operation that involves a dehydration step [3, 4]. Fruit pulp-based fruit leathers are nutritious and organoleptically acceptable to customers. They contain substantial quantities of dietary fibers, carbohydrates, minerals, vitamins, and antioxidants (which remain constituents of the finished product) [2, 5].

Most fresh fruits have a short harvest season and are sensitive to deterioration and even when stored under refrigerated conditions; therefore, making fruit leather from fresh fruits is an effective way to preserve fruits [4]. Fruit leathers are manufactured by dehydrating a fruit puree into a leather-like sheet [1]. Moisture is removed from the wet purees, which are usually laid on a large flat tray until the fruit puree or a prepared boiled fruit juice with additives changes into cohesive “leathery” sheets [6]. Fruit leathers are often considered as a health food and health food marketing images such as “pure,” “sun-dried,” or “rich in vitamins” are used to describe them [7]. There are large numbers of fruit leather products available on the market, such as mango leather, apricot fruit leather, grape leather, berry leather, kiwifruit leather, and jackfruit leather. In addition, mixed fruit leathers like guava and papaya fruit leather are also available.

Basically, fruit pulps are mixed with appropriate quantities of sugar, pectin, acid, and colour and then dried into sheet-shaped products. Gujral and Brar [5] added sugars and pectin to mango leathers. The sugar gave the product a sweeter taste and increased the solids content; then pectin was used to thicken the pulp, modify the flexible texture, and ensure the retention of the shapes of the dried product. Furthermore, they also prepared mango leather with the addition of potassium metabisulphite to get better sensory qualities and the results were satisfactory for customers/consumers. Chan Jr. and Cavaletto [8] made papaya leathers with sucrose and sodium bisulfite (SO_2_). They found that SO_2_ reduced the change in color of the papaya leathers during processing and storage. Various additives can be used, such as glucose syrup, sodium metabisulphite, and sorbic acid, depending on the types of fruit leather [9–11].

Sun drying is the simplest method of drying foods. Raisins, sultanas, and dried apricots are examples of the most popular sun-dried fruits. Sun drying permits the final product to have a translucent appearance, a normal color, and a gummy texture. However, there are disadvantages, such as a long drying process-exposure of the products to environmental contamination, dependency on weather conditions, and hand labor requirements. Therefore, alternative drying methods were developed to overcome the problems of hygiene and time, as these methods are rapid, safe, and controllable [4]. Modern dryers, such as tunnel dryers and forced air circulation cabinet dryers, have been used for making fruit leathers with a better color and flavor. Over 85% of industrial dryers are of the convective type with hot air or direct combustion gases used as the drying medium. The product changes during drying include shrinkage, puffing, and crystallization. Sometimes there are also desirable or undesirable chemical or biochemical reactions occurring that will cause changes in color, texture, odor, and other properties in the final product. Drying occurs from vaporization of the liquid by supplying heat to the wet material. Conduction, like contact or indirect dryers, convection such as direct dryers, and radiation or volumetrically by placing the wet material in a microwave or radio frequency electromagnetic fields are various methods that are used in making fruit leathers. The methods chosen are dependent on what kind of fruit and the commercial conditions. In many processes, incorrect drying methods result in irreversible damage to the quality of the final product which makes the product nonsaleable [3, 5]. With modern dehydrators and well-designed drying methods, fruit leathers can be dried at any time of the year to reach the requirements of customers.

In the current growing market of fruit leathers, commercial packaging is necessary. Packaging materials for fruit leather are required to prolong the shelf-life of the product and, normally, relate to the stability of water activity, microbiological stability, sensory properties, and physicochemical characteristics [12].

This paper/review will consider various researches on fruit leathers including the methods of preparation, the effect of drying conditions (e.g., temperature and velocity), and packaging and storage.

## 2. Methods of Preparation

**Figure 1:**
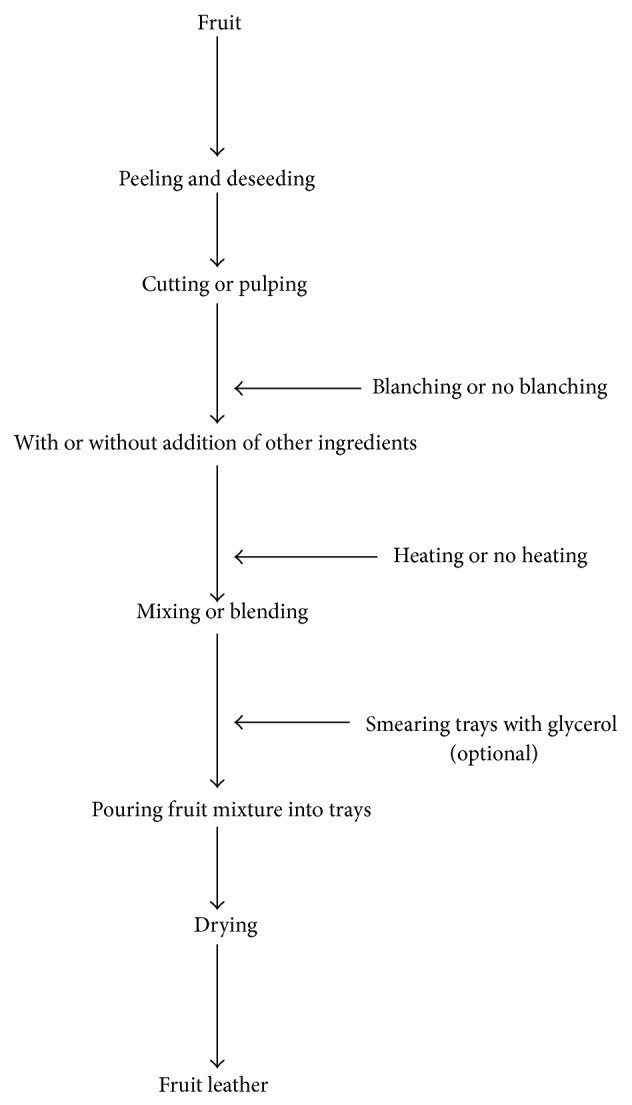
Schematic diagram of the general process for the production of fruit leather.

The general process of making fruit leather involves the preparation of the fruit puree, with or without addition of other ingredients before mixing and then drying (Figure 1). These processes may vary depending on the fruit used, the nature of the additional ingredients, and the drying method and technology.  Table 1 shows the advantages and disadvantages of the method of preparation of the different fruit leathers. As the results show, most fruit leathers have few disadvantages which are mostly on the lack of preservatives to protect the color.

### 2.1. Apple Leather

Apple leather was made using a puree made from golden delicious apples that was poured into 51 × 76 × 1.1 cm metal trays to a depth of 0.95 cm and then dried using 107°C air blowing across the trays at 4.6 m/s for 3.1 hours or 7.6 m/s for 2.7 hours [5].

Leiva Díaz et al. [13] processed apple leather from Granny Smith variety apples. Fruits were washed, cut into halves, cored, cut into 14 mm dices, and then steam-blanched for 600 s to avoid enzymatic browning, to soften the tissues, and to allow pectins to be dissolved and distributed before gelation. Seventy-nine g of blanched apple puree was added to 18 g of sucrose and 3 g of an aqueous solution of citric acid (0.174% w/w) per 100 g of formulation before drying to enhance the pectin-sugar-acid gelation. The apple puree mixture was poured into acrylic trays (0.18 m long by 0.138 m wide by 0.013 m high) and then dried in a hot air dryer at 60°C from an initial moisture content of 70.6% down to 26.9% w.b. in three hours. Apple leather was also prepared from a base formulation and procedure developed by Leiva Díaz et al. [13] but with added distilled water (23.1% w/w) and potassium metabisulphite (0.0057% w/w) [10]. The apple puree mixture was dried in a hot air dryer at a temperature of 60°C and an air velocity of 2 m/s to a final moisture content of 24.85% w.b. Once the dehydration process was finished, the gels were hermetically packaged and stored. A metabisulphite concentration of 173.7 mg/kg (based on the final product composition) was required to attain 100 mg/kg final product of SO_2_ in the final product.

Demarchi et al. [9] processed apple leathers at different temperatures. The formulation was similar to that reported by Leiva Díaz et al. [13] but added with the addition of polydextrose powder (9.0% w/w) and sucralose micronized powder (0.02% w/w). Another formulation was also prepared but with an additional potassium metabisulphite (0.0063% w/w). The apple puree mixtures were placed in 0.20 m square stainless steel trays, with an initial thickness of 6 mm and dehydrated in a tray dryer at 50, 60, and 70°C with an air velocity of 2 m/s. Samples were dried until reaching a moisture content of about 0.3 kg water/kg dry matter.

Aerated apple leathers were made by Valenzuela and Aguilera [14]. They used canned apple puree and gelatin (as a foaming agent). Apple puree and gelatin were weighed to give final concentrations of 0, 0.5, 1.0, and 1.5% (w/w) gelatin. The required amount of water was added to maintain the total solids content (21.5% w/w) constant in all formulations. The apple puree mixture was spread as a thin layer on the space of a 2 mm high frame that was placed in an aluminum tray; the puree was then levelled using a glass rod. The aluminum tray was previously covered with a silicone sheet to prevent the apple leather from sticking after drying. Drying was carried out in a cabinet dryer using a hot air at a temperature of 60 ± 1°C that reduced the moisture content from 3.65 kg water/kg dry solids to a final moisture content of about 0.13 kg water/kg dry solids.

### 2.2. Apple-Blackcurrant Leather

Fruit leather made from apple pulp, apple juice concentrate (AJC), blackcurrant concentrate (BCC), and pectin powder was prepared. Different levels of AJC (20, 30, and 40%), BCC (3, 6, and 9%), and pectin powder (0, 2 and 4%) were used to obtain various fruit leather products to determine the effects of the three factors on various physicochemical and nutritional qualities. About 315 g of the puree mixture was poured into aluminum pans with a nonstick surface (1 cm × 20 cm × 30 cm). The samples were then dried in a hot air dryer at 70°C with an air velocity of 0.20 m/s for 16 hours. The use of 62% apple pulp, 30% AJC, 6% BCC, and 2% pectin powder produced an acceptable fruit leather [15].

### 2.3. Apple-Apricot Leather

Bains et al.[16] processed apple-apricot leather using a fruit puree consisting of 82% apple puree, 16.5% apricot puree (as flavor component), and 1.5% apple juice concentrate. The fruit puree was poured in galvanized steel trays measuring 12.5 × 12.5 × 1.2 cm. The fruit puree was then placed in a pilot cabinet dryer at 85°C, with a flow rate of 4 m/s and relative humidity of 5% for 6.1 hours and this resulted in a good quality product. A two-stage operation with two hours of initial drying at 102°C followed by finish drying at 85°C for 3.5 hours also gave a good quality product.

### 2.4. Apricot Leather

A study was carried out to standardize the technology used for the preparation and storage of a wild apricot fruit bar [11]. The authors prepared the apricot bar by sorting and washing the fruit and then heating for 5 to 7 minutes in a stainless steel pan with water (100 mL/kg of fruit), before passing the fruits through a pulper to extract the pulp. The pulp was boiled over a low flame until its volume reduced to half and then mixed with different quantities of sugar (40%, 50%, and 60%). Fifty g of sugar was retained for mixing with the pectin to ensure a uniform distribution. Weighed quantities of pectin (0.20%, 0.30%, and 0.40%) were mixed by uniform sprinkling and continuous mixing. The treated mixture was then poured into aluminum trays (smeared with butter) in layers about 4-5 mm thick and the trays were placed in a mechanical dehydrator at 55 ± 2°C for about 6 hours.

### 2.5. Durian Leather

Che Man et al. [17] made a study on the effect of different dryers and drying conditions on the acceptability and physicochemical characteristics of durian leather. They prepared the durian leather from durian aril. The durian aril was blanched in an enclosed water bath for 5 minutes and then blended with the addition of 10% glucose syrup solid, 5% sucrose, water, 2.67% hydrogenated palm oil, and 0.45% soy-lecithin; 100 ppm egg yolk was added as a colorant. The mixture was formed into 1.2 mm thick sheets and dried in a forced-air cabinet dryer at a particular temperature. The time of drying was dependent on the combination used. Irwandi et al. [12] also processed durian leather from durian aril by blanching in a water bath at 85–100°C for 5 minutes and then blended with the same amounts of added ingredients as in Che Man et al. [17] except that 200 mg/kg sorbic acid was added as a preservative. All the mixture were formed into 1.2 mm thick sheets and then placed in either oven or cabinet dryers for dehydrating. It took 12.6 hours to dry at 50°C in the oven and 10 hours at 52.5°C in the cabinet dryer. Jaswir et al. [18] studied the effect of the addition of glucose syrup solid, sucrose, hydrogenated palm oil, and soy-lecithin on the sensory acceptability of durian leather. They developed two formulation stages. In Stage 1, durian aril was blanched by steaming at 85°C in an enclosed water bath for 5 minutes and then blended. The additive ingredients and formed sheets were the same as in Irwandi et al. [12]. The sheets were dried in a forced-air cabinet dryer at 47°C for 8 hours with 1.5 m/s air flow and then rolled into sheets. The most acceptable combination from Stage 1 was then chosen and used in Stage 2. In this stage, further hydrogenated palm oil (0–5%) and soy-lecithin (0–1.5%) were added during blending and the results showed that soy-lecithin has a significant effect on the aroma, appearance, and overall acceptability and a highly significant effect on taste acceptability; as hydrogenated palm oil affected the taste and aroma acceptability.

### 2.6. Guava Leather

A new process of making guava fruit leather was reported in Vijayanand et al. [19]. The guava leather was prepared by washing ripe guava, then crushing, and extracting them through a pulper to get a puree. The pectolytic enzyme Rohapect D5 L was added to the guava puree at a concentration of 0.5 mL/kg and then the puree was incubated at 40°C. After 2 hours, guava juice was obtained by pressing the puree and this was then mixed with maltodextrin, sucrose, soluble starch, wheat flour, pectin, and an antibrowning agent until it reached total soluble solids of 25°Brix. The mixture was then spread on stainless steel trays smeared beforehand with glycerol at the rate of 12 kg/m^2^ and was then dried at 50°C, 12% RH in a cross-flow hot air dryer with a 2.5 m/s flow rate to a final moisture content of 14 to 15%.

Babalola et al. [20] studied the effect of cold temperature storage on the quality attributes of guava fruit leathers. They made the guava leather by peeling the fresh guava and adding 20% sugar, 0.2% citric, and 0.1% sodium benzoate until the concentration of the pulp was 80%. The treated pulp was boiled, cooled, and spread on trays that were previously oiled with glycerol. The pulp was dried at 60°C for 8 hours.

In a study of the storage stability of guava leather in different packing materials by Kumar et al. [21], healthy and disease-free guava fruits were picked up for making guava fruit leathers. The guava fruits were washed, peeled, and pulped by passing the slices of fruit through a superfine pulper cum finisher. The pulp obtained was heated to 85°C to inactivate the enzymes and then cooled to about 45°C. Potassium metabisulphite (0.2%) was then added and mixed in. The treated pulp mixture was then poured into stainless steel trays previously smeared with glycerol and dried in cross-flow cabinet dryer at 60°C until the moisture level of the pulp reached 15–20%.

### 2.7. Guava-Papaya Leather

Blended guava-papaya leathers were made by mixing the pulps of guava and papaya in different ratios [22]. Both papaya and guava were washed, peeled, and chopped into pieces. The seeds of papaya were discarded and the fruit pieces were crushed in a mixer to make papaya pulp. The guava pulp was prepared by passing guava slices through a superfine pulper cum finisher. The blended papaya and guava fruit pulps were mixed in ratios of 80 : 20, 60 : 40, 40 : 60, and 20 : 80. The brix and acidity of all the blends were adjusted to 25°Brix and 0.5%, respectively. The pulp mixture obtained was heated to 85°C to inactivate the enzymes and cooled to about 45°C. Potassium metabisulphite (0.2%) was also added as a preservative before; the mixture was poured as a 1.00 cm thick layer in stainless steel trays previously smeared with glycerol and dried in a cross-flow cabinet dryer at 60°C until the final moisture of the product reached 15–20%.

### 2.8. Jackfruit Leather

Unfertilized floral parts of jackfruit were used to make jackfruit leather by Che Man and Sin [23]. Pieces of the unfertilized floral parts were placed in a jacketed kettle and cooked in boiling water for 60 minutes. The cooked unfertilized floral parts were then macerated into a puree by using a sharp knife blender and then 15% glucose syrup, 25% sugar, 5% water, 500 *μ*g, g^−1^ sodium metabisulphite, and 200 *μ*g/g sorbic acid were added to the puree. The mixture was placed into a 2 mm thin layer on aluminum foil and dried in a cabinet dryer at 50°C with an air velocity of 1.6 m/s for 24 hours.

Chowdhury et al. [24] studied the mathematical modelling of thin-layer drying of jackfruit leather. They prepared the jackfruit leather by extracting the juice from the jackfruit bulb and making it fibre-free by passing through a clean cloth and applying hand pressure. The jackfruit leather was dried in a thin layer (5 mm thickness) at temperatures ranging from 40 to 70°C. The relative humidity range was 20–70% and air velocity ranged from 0.5 to 3.0 m/s.

The effect of solar drying on the quality and acceptability of jackfruit leather made from fresh ripe whole jackfruits was evaluated by Okilya et al. [25]. They pretreated the jackfruit leather by cutting the fruits into half longitudinally, carving out the sticky central cores with a sharp knife, scooping out the bulbs by hand, and cutting out the ends of the bulbs to remove the seeds. The bulbs were chilled to retard enzymatic softening and microbial growth before further processing. They blended the pulp using a kitchen blender and put the mixture in a pan where it was concentrated for 15 minutes in a water bath at 70°C. The drying time was reduced because the concentration step evaporated off some of the water. Under a natural convection condition, the treated concentrates were cooled to room temperature and then formed into sheets on fabricated stainless steel metallic trays (20 cm × 20 cm × 3 mm) lined with waxed paper. These sheets were dried in a solar dryer (average temperature of 36.7°C for three days), convection oven dryer (50°C for 18 hours), and electric cabinet dryer (65°C for 6 hours with an air velocity of 1.7 m/s per square meter tray area) to reach moisture contents of about 18.50%, 14.79%, and 18.85%, respectively.

### 2.9. Kiwifruit Leather

Thin layers of pulped kiwifruit flesh under conditions of small Biot numbers were dried by Chen et al. [26]. The pulped kiwifruit flesh was dried in an oven dryer at temperatures from 35–70°C until very close to the equilibrium moisture content. The drying tray was of 1 mm thick aluminum plate and the shallow dish dimensions were 100 × 100 × 7 mm.

Vatthanakul et al. [7] studied gold kiwifruit leather product development using quality function deployment approach. The basic ingredients of this fruit leather were pectin powder 150° (1.00, 2.00, and 3.00 g per 100 g kiwifruit puree), sugar (6 g), salt (0.5 g), citric acid (0.2 g), water (10.0 g), and glucose syrup (10.0, 15.0, and 20.0 g). Nine formulations of fruit leather were produced using different combinations of pectin and glucose. Fruit puree and glucose syrup were mixed in a blender for 2 minutes before adding the other ingredients. The ingredients were mixed for additional 2 minutes and immediately spread onto stainless steel drying trays when the blend was consistent. They prepared the gold kiwifruit leather by evenly spreading the fruit puree (400 g) over stainless steel drying trays. The stainless steel trays were placed with a polyethylene bag to prevent the fruit leather from sticking to the trays after drying. They dried the gold kiwifruit leather by using hot air at 70 ± 1°C for 12 hours in a batch tray dryer, which had been preheated at least 0.5 hours before drying.

### 2.10. Longan Leather

Jaturonglumlert and Kiatsiriroat [27] prepared longan leather by combining convective and far-infrared drying systems. They made longan leather by uniformly spreading 100 g longan puree in a tray and placed it in a drying chamber. The air-velocity at the drying chamber was between 0 and 4.5 m/s and the temperature ranged from 30 to 80°C. A far-infrared ceramic heater with an intensity level control inside the chamber was used in heating the air. The sample for drying was kept in a tray under this IR heater. The sample for radiant heating was prepared by inserting a K-type thermocouple into the bottom of the puree layer. The combined convective and far-infrared drying experiment was conducted at five radiator temperatures (from 300°C to 500°C) with distance between the sample and the infrared heat source of 10–30 cm. The inlet air temperature and velocity were kept at 30°C and 0.5 m/s, respectively. The final moisture content of the sample was 14% (d.b.).

### 2.11. Mango Leather

A study on the sorption isotherms of fortified mango bars prepared from the puree of soft ripe mangoes was reported by Mir and Nath [28]. They prepared the mango bars by washing and peeling the mangoes, then pulping and heating the pulp at 91–93°C for 2 minutes. They added powdered cane sugar, 0.6% citric acid, and 1734 ppm potassium metabisulphite; the total solids of the mango puree were raised to 30%. The mango puree was spread uniformly on aluminum trays and dried for 14–16 hours in a cross-flow cabinet dryer at 63 ± 2°C. Azeredo et al. [29] studied the effect of drying and storage time on the physicochemical properties of mango leathers. They made the mango leather with mango puree passed through a 1 mm sieve and then spread onto Petri dishes. The drying was carried out in an oven at 60–80°C until the moisture content of the mango leather reached 15–18%.

The effect of skim milk powder, soy protein concentrate, and sucrose on the dehydration behavior, texture, color, and acceptability of mango leather was studied by Gujral and Khanna [30]. The mango leather was prepared by washing and peeling the mangoes and passing them through a pulper to obtain a mango pulp with 10.6% total solids. The pulp was blanched at 80°C for 5 minutes and then cooled. Potassium metabisulphite (0.2% w/w) was added to the pulp when it was cooling and then the pulp was sealed in glass jars and stored at 4°C. The mango leather was then dried in aluminum trays measuring 25.5 cm × 13 cm and 2 cm deep in a cabinet dryer at about 60°C and with an air velocity of 3.5 m/s. Soy protein and skim milk powder and sucrose were added to the mango pulp. They found that this significantly reduced the drying rate of mango leather and lowered the extensibility and energy. They determined that mango leather containing 4.5% skim milk powder and 4.5% sucrose was the most acceptable to the sensory panelists. In another research, Gujral and Brar [5] studied the effect of hydrocolloids on the dehydration kinetics, color, and texture of mango leather. They made the mango leather by passing the mango pulp through a pulper to obtain total solids of 14.3%. The pulp was blanched at 80°C for 5 minutes and then cooled. Potassium metabisulfate (0.2% w/w) was added during cooling. Sugar (20%) was added to increase its sweetness and total soluble solids. Hydrocolloids were also added to the mango pulps. The treated mango pulp was placed on aluminum trays measuring 25.5 cm × 13 cm and 2 cm deep and dried at a temperature of 60 ± 1°C and relative humidity of 15% in a cabinet dryer. The hydrocolloids added were found to decrease the drying rate during the initial 2 hours but had no significant effect later. Pushpa et al. [31] also evaluated the effect of incorporating defatted soy flour to process nutritionally enriched mango fruit leather by microwave drying. Sugar (50 g), corn flour (5 g), lime juice (2 g), roasted defatted soy flour with a protein content of 51.8%, and skim milk powder in the ratio of 1 : 1 were added to the mango pulp at concentrations of 10%, 15%, 20%, and 25%. They heated the treated mixture at each concentration to 80°C for 15 minutes and then dried the mixture in a microwave dryer. Drying was conducted at different power levels (2, 4, 6, 8, and 10, corresponding to 4, 8, 12, 16, and 20 w/g of sample) with a power cycle of 30 s on and 30 s off, respectively, until the sample reached a moisture content of 12–15%.

### 2.12. Papaya Leather

The dehydration and storage stability of papaya leather was investigated by Chan Jr. and Cavaletto [8]. The papaya leather was prepared by steaming whole papaya for 1 minute, slicing, and then separating the flesh, skin, and seeds. They pulped the treated fruits and acidified them until the pH was 3.5. After inactivating the enzymes by heating the puree, the puree was stored frozen at −18°C. Sugar (10% w/w) at 4.9 kg/m^2^ (11 b/ft^2^) was added in the papaya puree and then the puree was poured evenly onto Teflon-coated pans or pans sprayed with a lecithin release agent. Sodium bisulfite was added to give low (552 ppm) and high (1105 ppm) levels of SO_2_ treatment. The purees were dried in a forced draft oven until they reached about 12-13% moisture content or a water activity of 0.50–0.52. Babalola et al. [20] also made pawpaw (papaya) leather by peeling the fresh fruits and adding 20% sugar, 0.2% citric acid, and 0.1% sodium benzoate to a concentration of 80% pulp. The pulp was then boiled, cooled, and spread on trays that had been previously oiled with glycerol and then dried at 60°C for 8 hours.

### 2.13. Pear Leather

Huang and Hsieh [3] studied the physical properties, sensory attributes, and consumer preference of pear fruit leather. They prepared pear leather in 18 different formulations homogeneously blending pectin (16%, 20%, and 24% w/w), water (4%, 6% and 8% w/w), and corn syrup (0%, 8% w/w) at different levels with pear juice concentrate. They mixed distilled water (23°C) into the preblended mixture of pear concentrate and corn syrup (both at 5°C) with a presanitized Sunbeam Mixmaster blender for 1 minute and then added pectin to prevent the formation of lumps. They blended every second 400 g batch of the final mixture for another 3 minutes and then poured the treated batch into clean plastic flat-bottomed 7 × 10^3^ mm^2^ containers. The weight in each container was about 35 g so the height of the fruit leather sheet could be about 2.85 mm. They left the containers on the bench at 23°C until the mixture became evenly distributed (approximately 1 minute). They made the final leathers by placing the containers of the mixture in a convection oven at 70°C for 8 hours, with an air velocity in 0.4 m/s.

### 2.14. Pestil (Grape Leather)

Maskan et al. [4] reported hot air drying and sun drying for preparation of pestil (grape leather). The pestil (grape leather) was made by washing the grapes to remove dirt, leaves, and foreign materials and then crushing and pressing them manually. The seeds were removed from the juice by filtration using a cheese cloth. Seven grams of natural earth (70% CaCO_3_) was added to the juice per litre to reduce the acidity and clarify the juice. The mixture was boiled for 3–5 minutes in order to inactivate the enzymes which cause colour changes. The foam formed on the surface of the juice during boiling was removed. The juice was then separated from the calcium tartrate precipitate by filtration and centrifugation to obtain the final clarified juice which had a pH of 7.6 and Brix of 20°. The total juice was divided into two parts. A 3/4 part of juice was boiled again for 30 minutes with continued stirring to obtain a concentrated juice with 40°Brix. A wheat starch-juice mixture (starch dissolved in a 1/4 part of juice) was added to the boiling juice before boiling for another 4 minutes until it reached a concentration of 4 g/100 g of starch in the total fresh clarified juice. The cooked grape juice-starch mixture was evenly spread on an 8 cm diameter disk of cloth to be dried under hot air drying conditions or direct sunlight. The concentrated grape juice mixture samples were dried until there was no weight change. For the sun-dried products, the samples were dried under direct sunlight for 14 hours.

### 2.15. Pineapple Leather

The physicochemical characteristics and sensory optimization of pineapple leather was studied by Phimpharian et al. [32]. They researched the effects of glucose syrup (2%, 4%, and 6%) and pectin (0.5%, 1.0%, and 1.5%) concentrations. They prepared pineapple puree by removing the stalk and rinsing each whole pineapple, then removed the skin, divots, and leafy crown, then rinsed the treated pineapple flesh with tap water, cut them into pieces, and chopped for 30 s into a puree. The puree was placed into plastic bags and then stored at −18°C for up to 2 weeks until used. They left the pineapple puree in a refrigerator overnight before being used the next day. Pineapple puree was heated at 85 ± 5°C while stirring with an automatic pot stirrer at a speed of 57 rpm for 15 minutes and then mixed with pectin, glucose syrup, sugar (fixed at 15%), and maltodextrin (fixed at 2%). The puree was heated and stirred for another 80 minutes to obtain pineapple paste. They fed every 500 g portion of pineapple paste through the cylinder (an inner diameter of 42 mm) located on the top of the leather forming machine, and pressed the paste into the extruder zone with a pneumatically driven ram at a pressure of two bars, and then extruded them through a die (27 mm width × 2.2 mm thickness) at a screw speed of 50 rpm to obtain a flat rectangular paste. The flat pineapple paste was placed on a conveyor belt lined with a polypropylene plastic sheet, cut, and then dried in a hot air dryer at 60°C for 10 hours to form the pineapple leather.

### 2.16. Strawberry Leather

Lee and Hsieh [33] conducted an experiment with strawberry fruit leather to investigate its thin-layer drying kinetics. They blended strawberry puree, corn syrup, pectin, and citric acid together in 200 : 40 : 2 : 1 ratios and then spread the mixture into thin layers on an aluminum weighing dish of 70 mm diameter. The thin layers (1.8, 2.7, and 3.6 mm thicknesses) were then dried in a convection oven at various temperatures (50°C, 60°C, 70°C, and 80°C). The drying times for the strawberry leather samples to reach the safe-storage moisture content of 12% (wb) varied from 80 to 600 minutes in terms of the different drying temperatures and sample thickness. They found that the drying rates increased as the sample thickness decreased from 1.8 to 3.6 mm.

## 3. Effect of Drying Conditions

### 3.1. Combined Convective and Far-Infrared Drying

Jaturonglumlert and Kiatsiriroat [27] studied the heat and mass transfer in combined convective and far-infrared drying of longan leather. Infrared heating for food drying has a high efficiency of between 80 and 90% and infrared radiation could be transmitted through water at short wavelength and be absorbed on the surface at long wavelength [34]. The dryer had a blower to supply air into the drying chamber. The entering air velocity at the drying chamber varied from 0 to 4.5 m/s. The temperature of the entering air could be controlled at 30–80°C by an electrical heater. The drying chamber was a rectangular duct and well insulated. There was a far-infrared ceramic heater with an intensity level control inside and its maximum power was 800 W. The target final moisture content of 14% (d.b.) was used in order to compare the drying rate curve time for hot air drying alone as well as for the combined convective and far-infrared drying. The hot air drying was conducted at 70°C and 1.0 m/s, and the combined convective and far-infrared drying was at 400°C; the distance between the sample and the infrared heat source was 20 cm. They concluded that the second method provided a shorter drying time to the target final moisture content due to its higher heat and mass transfer coefficient.

### 3.2. Hot Air Drying

Demarchi et al. [9] studied the effect of different temperatures (50, 60, and 70°C) on the hot-air drying rate and retention of antioxidant capacity (AC) in apple leathers with and without potassium metabisulphite. The drying kinetics of apple leather were accurately predicted by a one-term diffusive analytical solution for plane sheets using internal-external control to predict mass transfer. The mass transfer Biot number was almost unity and the Arrhenius dependency of the effective diffusion coefficient with temperature provided an activation energy for drying of 20.6 kJ/mol. Retention of AC in the apple leathers was low (6–16%) and decreased for increasing air temperatures even when the resulting drying times were shorter. In mathematical terms, this effect is explained by the higher activation energy for AC losses (above 31 kJ/mol), compared with that for drying.

Thin-layer drying experiments of jackfruit leather were conducted by Chowdhury et al. [24]. The drying air was supplied by a centrifugal fan, through a galvanized iron (GI) pipe fitted with an orifice plate to the bottom of a metal tower packed with plastic rings. The heated air passed through the GI pipe to the overflow drying chamber. They found that an air temperature of 50°C was the optimum temperature for drying jackfruit leather and the moisture diffusivity increased from 3.25 × 10^−10^ m^2^/s at 40°C to 1.0062 × 10^−9^ m^2^/s at 70°C. The activation energy for moisture diffusion was 31.49 kJ/mol.

Hot air drying was used to make pestil (grape leather) by Maskan et al. [4]. The hot air drying experiments were conducted in a pilot plant tray dryer. The sample was dried from one side with hot air flowing parallel to the surface of the sample. The air velocities were 0.86 ± 0.03, 1.27 ± 0.04, and 1.82 ± 0.09 m/s and the sample thicknesses were 0.71 ± 0.035, 1.53 ± 0.070, 2.20 ± 0.110, and 2.86 ± 0.071 mm. In this study, the hot air drying temperatures were 55°C, 65°C, and 75°C (dry bulb) and 27°C, 30°C, and 33°C (wet bulb) temperatures, respectively. They found that the time required to reduce the moisture content to about 0.12 kg H_2_O/kg DS (11% wet basis) varied from 40 to 240 minutes, depending on drying temperatures and sample thickness. They determined that increasing the temperature at a constant sample thickness could reduce the time required to reach the equilibrium moisture content.

The effect of sun drying on color change of pestil was determined by Maskan et al. [35]. The Hunter a-value of pestil increased from 3.50 to 3.74 during the first stage of sun drying (0 to 325 minutes) and increased slowly from 3.74 to 3.78 (325 to 1830 minutes). However, Hunter L- and b-values showed fluctuation during drying without a constant trend. The order of the reaction for color change based on the a-value during sun drying was a zero order.

Che Man et al. [17] studied the effect of different dryers and drying conditions on the acceptability and physicochemical characteristics of durian leather. In the oven drying experiment, they found that the most acceptable taste could be achieved by drying at 52.24°C for 11.63 hours, and for the texture it was 52.5°C for 9.00 hours. The best conditions for aroma and appearance were 50.63°C for 12.00 hours and 51.7°C for 12.58 hours, respectively. For overall acceptability, the most acceptable combination was 50°C for 12.75 hours. In the forced-air cabinet drying experiment, they found that the most acceptable conditions for taste, texture, and aroma were 52.42°C for 10.42 hours, 47.50°C for 10.00 hours, and 49.71°C for 13.50 hours, respectively. The best conditions for appearance and overall acceptability were 53.81°C for 7.71 hours and 52.50°C for 10.00 hours, respectively. They found that drying in a cabinet dryer to reach a certain moisture level took less time than in the oven at the same temperature. For example, drying at 60°C for 7 hours gave a product with 15% moisture from the oven but less than 13% moisture from the cabinet dryer.

Chan Jr. and Cavaletto [8] studied the dehydration and storage stability of papaya leather. The papaya puree was dried in a forced draft oven at an air velocity of 110 fpm to reach about 12-13% moisture content or water activity of 0.50–0.52. The average drying times needed for 74°C, 84°C, and 94°C were 4.5, 3.9, and 3.1 hours, respectively.

### 3.3. Microwave Drying

Microwaving significantly reduces the drying time for dehydrating a product. Microwave dries a food material faster than a conventional dryer does using short high-frequency energy waves similar to TV, radar, and radio waves. Mango fruit leather was prepared using a 750 W, 2450 MHz microwave by Pushpa et al. [31]. The results showed that the drying time was reduced from 200 seconds to 60 seconds as the microwave power level was increased from 2 to 10, corresponding to 4 to 20 W/g, respectively. Therefore, mass reduction of the sample was rapid at higher microwave power level and the drying time was very short. They also concluded that decreasing the microwave power level may increase the drying time to unacceptable levels.

### 3.4. Solar Drying-Cabinet Drying

A cabinet dryer is a direct solar drying system. Part of the incidence solar radiation on the glass cover is transmitted inside the dryer while the remaining is reflected back to the air. Then part of the transmitted radiation is then reflected back from the surface of the fruits while the remaining part is absorbed by the surface of the fruits. The temperature of the fruit products increases and they start emitting long wavelength radiation as they absorbed the solar radiation. The long wavelength radiation emitted was not able to escape to the atmosphere because of the presence of the glass cover. The glass cover reduced the direct convective losses to the ambient air, which further benefitted the rise in temperature of the fruit products and chamber temperature, respectively. Convective and evaporative losses occur inside the chamber from the heated product. Moisture is removed by the air entering the chamber from below and escaping through another opening provided at the top [35].

Sharma et al. [36] investigated three different types of solar dryers based on the principle of natural as well as forced convection drying conditions. They reported that the cabinet-type solar dryer is very well suited to drying a small quantity of fruits and vegetables on a domestic and household scale.

### 3.5. Solar Tunnel Drying

Chowdhury et al. [37] also made jackfruit leather using solar tunnel drying under the same weather conditions as Chowdhury et al. [24] in the previous section. The solar tunnel dryer was a forced convection mixed-mode solar dryer. It consisted of a flat plate air-heating collector, a drying unit, a solar module of 40 W capacity, and two 152 mm diameter 12 V DC fans to provide the required airflow over the product. The air-heating collector and the drying unit were connected in series. The collector was painted black so the absorption of solar radiation can be improved. Both of the collector and drying area were covered with a 0.2 mm thick transparent UV stabilised polyethylene sheet. The input of solar radiation on the collector and on the dryer section comprised the input solar radiation into the solar tunnel dryer. The ambient air temperature during drying varied from 30°C to 36°C while the collector outlet temperature varied from 43°C to 58°C. During the drying period, the average temperature in the collector outlet was 54.2°C and the average rise in temperature was 19.16°C. Jackfruit leather was dried to an 11.88% moisture content (w.b.) from an initial moisture content of approximately 76% (w.b.) after two days of drying in the solar tunnel dryer. It has been found that the energy efficiency of the collector varies from 32.34 to 65.30%. The variation of solar radiation varies between 100 and 600 W/m^2^.

The experimental and neural network prediction for the performance of a solar tunnel dryer for drying jackfruit bulbs and leather was studied by Bala et al. [38]. The structure of the solar tunnel dryer used was similar to the one used in Chowdhury et al. [37]. Glass wool was added as an insulation material to reduce heat loss from the dryer. The cover was fixed like a sloping roof to prevent the entry of water inside the dryer unit when it rained. The air at the required flow rate was provided by two direct-current fans operated by one photovoltaic module. The solar radiation passed through the transparent cover of the collector and heated the absorber. The solar tunnel drying required 14 hours to dry jackfruit samples from 78.12 to 5.03% moisture content or 19 hours from 82.44 to 9.77% moisture content.

### 3.6. Sun Drying

Solar energy is used for drying products. When short wavelength solar energy falls on the uneven product surface, a part of it was absorbed by the product's surface while the rest was reflected back. The amount of energy absorbed depends on the color of the product. The absorbed radiation was then converted into thermal energy and increased the temperature of product. There were also long wavelength radiation losses from the surface to the ambient air. In addition, convective heat losses that occurred were due to the wind blowing moist air over the product surface. The moisture contents reduced from these evaporative losses and so the fruit products were dried [34].

Maskan et al. [4] dried pestil (grape leather) using sun drying. The samples were exposed directly to sunlight for 14 hours with an intensity of sunlight at 140 ± 62 J/cm^2^ min during the day. The percent relative humidity of the air was 43.5 ± 11.4; the temperature by day was 21.4 ± 9.2 and by night 14.9 ± 5.3°C; the air velocity was 0.53 ± 0.34 by day and 0.32 ± 0.12 m/s by night. The time used to reach the moisture content of 11% (wet basis) was 3, 5, 15, and 25 hours, depending on the thickness of the product. They found that the thinner the sample was, the faster it dried.

Jackfruit leather was made by Chowdhury et al. [37] under natural sun drying conditions. The jackfruit leather was dried to 13.8% (w.b.) under open sun drying for two days. However, the drying samples received energy only from incident solar radiation and lost a significant amount of energy to the environment.

## 4. Effect of Packaging and Storage

Kumar et al. [21] prepared guava leather to study its storage stability in four packing materials: polypropylene (PP), butter paper (BP), metalized polyester polyethylene (MPP), and aluminum foil (AF) under ambient and low temperature conditions (10  ±  1°C). During storage, the moisture content of the product decreased significantly under ambient conditions while it increased slightly under low temperature conditions. The products packed in MPP and AF showed the minimum loss of moisture. During storage period, it was also found that the acidity of guava leather increased more than under low temperature conditions. A 40–50% reduction in vitamin C was observed under ambient conditions irrespective of the packaging material. The storage of guava leather in different packing materials invariably increased the reducing sugar content during storage under low and ambient temperatures. It was concluded that the samples stored in MPP retained a higher percentage of nutrients and minimum microbial counts at the end of storage under both conditions. The organoleptic rating was also higher for the samples stored in MPP. Polypropylene could not be compared with MPP or AF wrappers but, after considering economic conditions, it could be used for a shelf-life of one to two months. However, even though AF packaging was easy to use, the development of pin holes during handling rendered it unsuitable for packaging the guava leather product.

The quality of apple leathers with and without potassium metabisulphite (KMBS) during storage was evaluated by Quintero Ruiz et al. [10]. A KMBS-added formulation satisfactorily maintained the quality characteristics of apple leathers without microbial development over a 7-month storage period. The browning index (BI) was observed to increase during storage at 20°C. This increase was especially moderate in the KMBS-added leather. A first-order kinetic model gave the best fit for the browning data. The antioxidant activity (AA), determined over storage and expressed as chlorogenic acid equivalents, decreased by 47% during the 7-month period at 20°C in the control formulation, while losses in the KMBS-added formulation were considerably lower, 15.9% of the initial value. An accelerated storage experiment of the KMBS-added formulation at 30°C allowed the estimation of the effect of storage temperature using a *Q*_10_ coefficient of 2.55 for BI and 16.3 for AA. According to these *Q*_10_ values, browning would be the storage-limiting parameter at or below 20°C.

The thermodynamic properties and sorption equilibrium of a pestil were studied by Kaya and Kahyaoglu [39]. They illustrated that water activity was one of the most important quality factors for long-term storage because the changing water activity directly affected all chemical and microbial deterioration reactions.

Kaya and Maskan [40] determined the water vapor permeability (WVP) of pestil (a fruit leather) made from boiled grape juice with starch at three different temperatures (15, 25, and 37°C) and different relative humidity (RH) values (31 to 76%). After exposure to the high RH environment, the thickness of the film was increased, showing the adsorption of water by the leather itself. It was found that the water vapor transmission rate (WVTR) and WVP of pestil were strongly affected by the changing RH and temperature. The effect of RH on WVP became more pronounced with increasing temperature.

Babalola et al. [20] evaluated the effect of cold temperature storage on the quality attributes of pawpaw (papaya) and guava leathers. The calorific content, water activity, pH, and total mould count in pawpaw leather were significantly higher than those in guava leather throughout the duration of storage. However, the guava leather had a higher texture. Sensory scores in relation to the period of storage showed that guava leather gave better results in overall acceptability at zero, one, and two months of storage at 8 ± 1°C. The guava leather was accepted better based on better sensory qualities in fruitiness, smell, chewiness, toughness, color, and overall acceptability when various cold storage temperatures were considered during storage.

The effects of types of packaging materials on the physicochemical, microbiological, and sensory characteristics of durian fruit leather during storage were studied by Irwandi et al. [12]. The study was carried out over 12 weeks at room temperature using four types of packaging materials: laminated aluminum foil (LAF), high-density polyethylene (HDPE), low-density polyethylene (LDPE), and polypropylene (PP) film. They found that LAF had the lowest decrease in water activity and changes in moisture content so that it could maintain the desired textural characteristic of fruit leather. Low-density polyethylene resulted in the highest changes in moisture and water activity. The type of packaging materials and storage time had significant effects on the nonenzymatic browning of durian leather with LAF having a lowest decrease in color quality while LDPE showed the highest degree of browning. Both the type of packaging materials and storage time affected the texture significantly by increasing its hardness during storage. The laminated aluminum foil maintained the desired texture well while samples packed in LDPE had the greatest increase in hardness up to eight weeks. The acidity level fluctuated during storage. The packaging materials and storage time both significantly affected microbial growth during storage. Laminated aluminum foil seemed to be the best material to inhibit growth of the mesophilic bacteria, moulds, and yeasts. However, the largest increase in microbial counts was in the LDPE. Panelists gave the lowest scores for texture, appearance, aroma, and overall acceptability to the LDPE-packed samples and the highest score to the LAF-packed samples. These results related to the water vapor characteristics of the packaging material. However, organoleptically, all four packaging materials were acceptable.

Vijayanand et al. [19] assessed the storage stability and packaging requirements of a guava fruit bar prepared using a new process that gave better texture and sensory properties. A mango bar was also prepared as a comparison in this experiment. The bars were packed separately in two materials: PP (polypropylene) and BOPP (biaxially oriented polypropylene). The polypropylene had a water vapor transmission rate (WVTR) of 6 × 10^−3^ kg/m^2^/d at 90% RH, 38°C and an oxygen transmission rate (OTR) of 35 × 10^−3^ L/m^2^/d atmosphere at 25°C. The biaxially-oriented polypropylene had a water vapor transmission rate of 4 × 10^−3^ kg/m^2^/d at 90% RH, 38°C and an OTR of 2.5 L/m^2^/d atmosphere at 25°C. Guava and mango bars had similar textural characteristics initially that reduced after three months of storage at ambient conditions. The nonenzymatic browning of both guava and mango bars increased significantly after 60 days of storage. The overall quality of both the guava and mango bars packed in BOPP and PP decreased significantly at the end of 60-day storage because the bars absorbed moisture. However, the guava and mango bars packed in pearlized BOPP or PP were sensorily acceptable with respect to color, flavor, texture, and overall quality for up to 90 days at 27°C and 65% RH and for up to 30 days at 38°C and 92% RH.

## 5. Conclusions

Fresh fruits are known to be excellent sources of vitamins, minerals, fibers, carbohydrates, and other bioactive compounds. Fruit leathers provide attractive, colored, and flavorsome products for people. A variety of researches have been carried out to study the effects on fruit leathers of different methods of preparation, different drying conditions, and packaging and storage conditions.

For the method of preparation, most fruit leathers were prepared by sorting, washing, peeling, and seed removing, and then cutting into slices which can be pureed or pulped easily. Purees are heated, boiled, or blanched in a water bath in order to inactivate the enzymes. Additives such as sugar, pectin, acid, glucose syrup, and color are often added before or during blending. The additives include potassium metabisulphite, sodium bisulfite, sodium metabisulphite, sucrose, soy protein and skim milk powder, corn syrup, and starch. These ingredients are mixed with the fruit puree to make fruit leathers with a higher quality, longer storage, or better organoleptic quality than the original fruit. Most fruit leathers are dried at 30 to 80°C, especially at 50 to 60°C for up to 24 hours or until they have reached the final moisture content of 12–20% (w.b.).

Various drying systems are used in making fruit leather depending on what fruits are being dried and how the products are designed. Combined convective and far-infrared drying provided a shorter drying time due to its higher heat and mass transfer coefficients. Hot air drying, including oven drying, forced-air cabinet drying, and thin-layer drying, is widely used and the time taken depended on the drying temperatures and sample thicknesses. Microwave drying reduced the sample mass rapidly and has a very short drying time. Solar drying included cabinet drying, solar tunnel drying, and sun drying. Solar cabinet dryers were well suited to drying small quantities of fruits; solar tunnel drying was a forced convection mixed-mode solar dryer which collected solar radiation from the atmosphere to input solar radiation into solar tunnel dryer. Sun drying is simple but lengthy and unhygienic.

Water activity was one of the most important quality factors for long-term storage. Laminated aluminum foil (LAF) gave the highest score for overall acceptability from panelists and low-density polyethylene had the lowest score for packaging fruit leathers. The product packed in metalized polyester polyethylene and aluminum foil showed low losses of moisture. Other packaging materials like high-density polyethylene, polypropylene, butter paper, and biaxially-oriented polypropylene were shown to be acceptable for storage of fruit leathers at low temperatures (8–10°C) for up to two months.

The data from this paper will be useful to many in the food industry and consumers who are health-conscious.

## Figures and Tables

**Table 1:** Advantages and disadvantages of the method of preparation for the different fruit leathers.

Method	Fruit	Advantages	Disadvantages
Gujral and Brar [5]	Apple	Simple since it only used the fruit puree	No other ingredients to improve the qualities and preservatives to protect the color
Leiva Díaz et al. [13]	Apple	Addition of other ingredients to improve pectin-sugar-acid gelation	No preservatives to protect the color
Quintero Ruiz et al. [10]	Apple	Addition of other ingredients to protect the color and improve the qualities	None
Demarchi et al. [9]	Apple	Addition of other ingredients to protect the color and improve the qualities	None
Valenzuela and Aguilera [14]	Apple	Porous apple leather	No preservatives to protect color
Bains et al. [16]	Apple-apricot	Addition of apricot to compliment the apple flavor	No other ingredients to improve the qualities and preservatives to protect the color
Diamante et al. [15]	Apple-blackcurrant	Addition of other ingredients to improve physicochemical and sensory qualities	Need to reduce drying time to produce a much better fruit leather
Sharma et al. [11]	Apricot (wild)	Utilization of wild apricot	No preservatives to protect the color
Che Man et al. [17]	Durian	Utilization of durian	Addition of nontraditional ingredients like palm oil, soy lecithin, and egg yolk
Irwandi et al. [12]	Durian	Utilization of durian and addition of preservative for storage stability	Addition of nontraditional ingredients like palm oil, soy lecithin, and egg yolk
Vijayanand et al. [19]	Guava	Utilization of guava	None
Babalola et al. [20]	Guava	Utilization of guava	No preservatives to protect the color
Kumar et al. [21]	Guava	Utilization of guava	None
Kumar et al. [22]	Guava-papaya	Addition of papaya to compliment the guava flavor	None
Che Man and Sin [23]	Jackfruit	Utilization of the unfertilized floral parts of jackfruit	None
Chowdhury et al. [24]	Jackfruit	Simple since it only used the fruit puree	No other ingredients to improve the qualities and preservatives to protect the color
Okilya et al. [25]	Jackfruit	Some pretreatments were done on the fruit puree to improve the quality	No other ingredients to improve the qualities and preservatives to protect the color
Chen et al. [26]	Kiwifruit	Simple since it only used the fruit puree	No other ingredients to improve the qualities and preservatives to protect the color
Vatthanakul et al. [7]	Kiwifruit	Addition of other ingredients to improve the qualities	No preservatives to protect the color
Jaturonglumlert and Kiatsiriroat [27]	Longan	Utilization of longan	No other ingredients to improve the qualities and preservatives to protect the color
Mir and Nath [28]	Mango	Addition of other ingredients to protect the color and improve the qualities	None
Azeredo et al. [29]	Mango	Simple since it only used the fruit puree	No other ingredients to improve the qualities and preservatives to protect the color
Gujral and Khanna [30]	Mango	Addition of other ingredients to protect the color and improve the qualities	None
Gujral and Brar [5]	Mango	Addition of other ingredients to protect the color and improve the qualities	None
Pushpa et al. [31]	Mango	Nutritionally enriched mango leather	Addition of nontraditional ingredients like corn flour, soy flour, and skim milk powder
Chan and Cavaletto [8]	Papaya	Addition of other ingredients to protect the color and improve the qualities	None
Babalola et al. [20]	Papaya	Addition of other ingredients to improve the qualities and storage stability	No preservatives to protect the color
Huang and Hsieh [3]	Pear	Addition of other ingredients to improve the qualities	No preservatives to protect the color
Maskan et al. [4]	Pestil (grape)	Utilization of grape juice	No preservatives to protect the color
Phimpharian et al. [32]	Pineapple	Addition of other ingredients to improve the qualities	No preservatives to protect the color
Lee and Hsieh [33]	Strawberry	Addition of other ingredients to improve the qualities	No preservatives to protect the color
